# A Toll/IL-1R/resistance domain-containing thioredoxin regulates phagocytosis in *Entamoeba histolytica*

**DOI:** 10.1186/1756-3305-5-224

**Published:** 2012-10-08

**Authors:** Ismael Mancilla-Herrera, Alfonso Méndez-Tenorio, Isabel Wong-Baeza, Alexis P Jiménez-Uribe, Marcela Alcántara-Hernández, Ramon Ocadiz-Ruiz, Mario A Moreno-Eutimio, Lourdes A Arriaga-Pizano, Constantino López-Macías, Jorge González-y-Merchand, Armando Isibasi

**Affiliations:** 1 Medical Research Unit on Immunochemistry, Specialties Hospital. National Medical Centre “Siglo XXI”. Mexican Social Security Institute (IMSS), Mexico City, Mexico; 2 Graduate Program on Biomedicine and Biotechnology, ENCB-IPN, Mexico City, Mexico; 3 Genomic Biotechnology and Bioinformatics Laboratory, Biochemistry Department, National School of Biological Sciences, National Polytechnic Institute (ENCB-IPN), Mexico City, Mexico; 4 Graduate Program on Immunology, ENCB-IPN, Mexico City, Mexico; 5 Mexico’s Juarez Hospital, SSA, Mexico City, Mexico; 6 Departamento de Infectómica y Patogénesis Molecular. Departamento de Patología Experimental, Centro de Investigación y de Estudios Avanzados del IPN, México City, México; 7 Molecular Microbiology Laboratory, Microbiology Department, ENCB-IPN, Mexico City, Mexico

**Keywords:** *Entamoeba histolytica* phagocytosis, Toll/IL-1R/resistance domain, Erythrocytes phagocytosis, Bacteria phagocytosis

## Abstract

**Background:**

*Entamoeba histolytica* is a protozoan parasite that infects humans and causes amebiasis affecting developing countries. Phagocytosis of epithelial cells, erythrocytes, leucocytes, and commensal microbiota bacteria is a major pathogenic mechanism used by this parasite. A Toll/IL-1R/Resistance (TIR) domain-containing protein is required in phagocytosis in the social ameba *Dictyostelium discoideum*, an ameba closely related to *Entamoeba histolytica* in phylogeny. In insects and vertebrates, TIR domain-containing proteins regulate phagocytic and cell activation. Therefore, we investigated whether *E. histolytica* expresses TIR domain-containing molecules that may be involved in the phagocytosis of erythrocytes and bacteria.

**Methods:**

Using *in silico* analysis we explored in *Entamoeba histolytica* databases for TIR domain containing sequences. After silencing TIR domain containing sequences in trophozoites by siRNA we evaluated phagocytosis of erythrocytes and bacteria.

**Results:**

We identified an *E. histolytica* thioredoxin containing a TIR-like domain. The secondary and tertiary structure of this sequence exhibited structural similarity to TIR domain family. Thioredoxin transcripts silenced in *E. histolytica* trophozoites decreased erythrocytes and *E. coli* phagocytosis.

**Conclusion:**

TIR domain-containing thioredoxin of *E. histolytica* could be an important element in erythrocytes and bacteria phagocytosis.

## Background

*Entamoeba histolytica* is the etiological agent of amebiasis. It is estimated that this protozoan parasite infects 500 million people worldwide (its prevalence is around 1% in industrialized countries and reaches 50–80% in tropical countries, causing 40,000–110,000 deaths per year) [1-3]. Phagocytosis of epithelial cells, erythrocytes, leucocytes and bacteria from the commensal microbiota is a major pathogenic mechanism used by *E. histolytica*. Phagocytosis requires recognition of ligands on target cells and activation of signaling pathways that lead to cytoskeletal reorganization and vesicle trafficking. In *E. histolytica*, the recognition of target cells is mediated by a galactose/N-acetylgalactosamine-binding lectin [4-6] and by a phagosome-associated transmembrane kinase (PATMK) that binds phosphatidylserine in host cells [7]. This is a mechanism that involves the recruitment of thiol-specific antioxidants (such as thioredoxins) for phagosome biogenesis and cytoskeletal rearrangement [8-10]. Included in thioredoxin functions are cell protection from oxidants, regulation of transcription factors and protein binding, and catalysis of protein folding [11,12].

Phagocytosis is essential for the survival of unicellular organisms. In the social ameba *Dictyostelium discoideum*, a Toll/IL-1R/resistance (TIR) domain-containing protein (TirA) is required for the phagocytosis of bacteria, which is essential for nutrition and for protection against infection [13]. Moreover, phagocytic cells play a central role in the innate immune systems of multicellular organisms, and TIR domain-containing proteins regulate the activation of these cells in insects and vertebrates. The TIR domain encompasses three highly conserved regions; i.e., Box1, Box2, and Box3 [14].

The TIR domain is present in a wide variety of eukaryotic organisms, from free-living amebas (which are closely related to *E. histolytica* in phylogeny) to insects and vertebrates. It has been suggested that in *E. histolytica*, the phagocytosis of natural substrates (such as human erythrocytes and *E. coli*) is mediated by TIR domain-containing proteins that participate in a signaling component that existed before the diversification of eukaryotes [15]. Therefore, in the present study, we investigated whether *E. histolytica* expresses TIR domain-containing molecules that participate in the regulation of the phagocytosis of human erythrocytes and *E. coli*. Using an *in silico* analysis of the *E. histolytica* proteome, we identified a TIR domain-containing sequence that corresponds to a thioredoxin. Furthermore, the downregulation of this thioredoxin by siRNA led to decrease of phagocytosis of erythrocytes and *E. coli* by *E. histolytica* trophozoites. These results suggest that the TIR domain-containing thioredoxin is involved in *E. histolytica* phagocytosis.

## Methods

### *In silico* analysis of the *E. histolytica* TIR domain-containing sequences

A Hidden Markov Model (HMM) for TIR domain proteins was built with HMMER software v2.3.2 ( http://hmmer.janelia.org) and a seed alignment collection of TIR proteins from PFAM database. (Pfam:PF01582, http://www.sanger.ac.uk/Software/Pfam) [16]. Through the HMM, a sequence analysis was implemented to search proteins that contain a probable TIR domain in Protein (NCBI), the Wellcome Trust Sanger Institute ( http://www.sanger.ac.uk), and the Pathema Bioinformatics Resource Center ( http://pathema.jcvi.org) databases for *Entamoeba* genus. Using BLAST (Basic Local Alignment Search Tool, http://blast.ncbi.nlm.nih.gov/Blast.cgi) on *E. histolytica* proteins of NCBI databases, sequences of primary structure scoring E values < 0.001 with TIR domain-containing proteins of *Entamoeba* species were selected as homologous proteins.

The primary and secondary structures of the TIR domain-containing proteins of *Entamoeba histolytica* identified were compared with the primary structures of the TIR domains of *Arabidopsis thaliana* (TAO1, GenPept: ABS82021), *Drosophila melanogaster* (Toll4, GenPept: AAF52747), and *Homo sapiens* (IL-1R, TLR2, and MyD88, GenPept: AAB84059, AAH33756 and AAC50954 respectively) by a multiple sequence alignment calculated with T-Coffee ( http://tcoffee.crg.cat/). Secondary structures were calculated with the Psipred Protein Structure Prediction Server ( http://bioinf.cs.ucl.ac.uk/psipred/). The tertiary structure of the TIR domain of the identified protein was modeled using I-TASSER server ( http://zhanglab.ccmb.med.umich.edu/I-TASSER/[17,18]) and compared with the tertiary structure of the TIR domain of human interleukin-1 receptor (PDB: 1T3GA). The best structural alignment was calculated by Chimera ( http://www.cgl.ucsf.edu/chimera/). The obtained structures were displayed in pdb format using RasMol v. 2.6.

### Culture of *E. histolytica* trophozoites

Trophozoites of the *E. histolytica* strain HM-1:IMSS were axenically grown in TYI-S-33 medium, according to Diamond et al. [19]. Trophozoites were grown at 37°C for 40–72 h and harvested by chilling on ice water for 10 min, to detach them from the culture tubes. Trophozoites were washed twice in phosphate-buffered saline solution using low-speed centrifugation (600 × g for 5 min) and suspended in TYI-S-33 medium to a final concentration of 10^6^ cells/mL.

### Reverse transcriptase (RT)-PCR assays

Total RNA was extracted from *E. histolytica* trophozoites using the TRIzol reagent (Invitrogen, Carlsbad, CA, USA). RNA was treated with DNase (Qiagen, Germantown, MD, USA) and reverse-transcribed using SuperScript II RNase H-Reverse Transcriptase (Promega, WI, USA). Primers for thioredoxin and PATMK (which was used as an expression control) were designed using Primer3 v.0.4.0 ( http://frodo.wi.mit.edu/primer3[20]) and actin primers were selected according to background [21]. The final reaction mixture contained 10 nM of each dNTP (Promega), 10 × Mg-free reaction buffer (Promega), 25 mM MgCl_2_ (Promega), 0.25 μl of dimethyl sulfoxide (Sigma, St. Louis, MO, USA), 2.5 U of Taq DNA polymerase (Promega), 0.5 μM of each primer (Thioredoxin: sense 5′–GGAGGTAATGGCTGAAATGC–3′, antisense 5′–GGAGGTAATGGCTGAAATGC–3′; PATMK: sense 5′–AATGGGTGTGCTGTTTGTCA–3′, antisense 5′–CCCTTCAGCACATCTGTCAC–3′; actin: sense 5-AGCTGTTCTTTCATTATATGC-3, antisense 5-TTCTCTTTCAGCAGTAGTGGT-3, and 100–1,000 ng of cDNA, in a final volume of 25 μL. The reaction mixture was denatured at 94°C for 5 min, followed by 35 cycles of denaturation (94°C for 60 s), annealing (59°C for 90 s), and extension (72°C for 90 s); a final extension of 5 min at 72°C was also performed. PCR products were electrophoresed in 2% agarose gels at 70 V for 60 min and stained with *GelRed*™ Nucleic Acid Stain (Invitrogen). The density band was documented by a EC3 Bioimaging System (UVP, CA,USA), using ultraviolet illumination. Densitometric analysis was made using VisionWorksLS program v6.4.3 (UVP, CA, USA).

### Silencing of gene expression

Expression of thioredoxin and PATMK were silenced using small interfering double stranded-RNAs ( Additional file 1: Table S1), which were designed using the SciTools for RNAi Design from Integrated DNA Technologies ( http://www.idtdna.com/Scitools/Applications/RNAi/RNAi.aspx). For each gene, three double-stranded oligonucleotides of 21–23 bp that bound to the initial, mid, and terminal regions of the corresponding transcripts were used. Each oligonucleotide (50, 100 and 250 μg) were added to 10^6^ trophozoites in a final volume of 1.0 mL of TYI-S-33 medium, and the trophozoites were cultured for 15 h at 37°C (“soaking strategy” [22]). The silencing efficiency was evaluated using the RT–PCR approach described above.

### Erythrophagocytosis assay

All experimental procedures were approved by the Local Ethical Committee of Health Research of Specialties Hospital, Mexican Social Security Institute (IMSS), Mexico City, Mexico (Register number. R-2009-3601-157). Venous blood (2 mL) from healthy donors was layered over Lymphoprep™ (Axis-Shield, Oslo, Norway) and centrifuged at 800 × *g* for 20 min, to remove mononuclear cells. Erythrocytes were washed and suspended in phosphate-buffered saline solution. Erythrocytes (1 × 10^7^) were incubated with 1 × 10^6^ trophozoites (untreated or in the presence of thioredoxin or PATMK silencing) in 1 mL of TYI-S-33 medium at 37°C for 30 min, with agitation at 150 rpm. One milliliter of phosphate-buffered saline solution was added after the incubation, and the culture plate was centrifuged at 600 × *g* for 5 min. The pellet was lysed in 1 mL of cold concentrated formic acid (Sigma-Aldrich), and absorbance at 400 nm was measured using a spectrophotometer (Beckman Coulter, DU® Series 700 UV/Vis Scanning Spectrophotometer). Results were analyzed using Repeated-measures one-way ANOVA and Bonferroni test. Significance was set at *P* < 0.05.

### Bacteria phagocytosis assay

The *Escherichia coli* strain DH-5α was transformed with a plasmid encoding GFP and an ampicillin resistance gene and grown overnight at 37°C in LB medium with 100 mg/mL of ampicillin. Bacteria were harvested by centrifugation at 9,000 × *g* for 10 min, washed three times with phosphate-buffered saline solution, and suspended in phosphate-buffered saline solution to a concentration of 1 × 10^8^ colony forming unit (CFU) per mL. Incubation of 1 × 10^8^ CFU with 1 × 10^6^ trophozoites (untreated or in the presence of thioredoxin or PATMK silencing) was performed in 1 mL of TYI-S-33 medium at 37°C for 45 min, with agitation at 150 rpm. Gentamicin (15 ng) was added after the incubation; 10 min later, the culture plate was centrifuged at 600 × *g* for 5 min. The pellet was suspended in 1 mL of phosphate-buffered saline solution, and the fluorescence of each sample was measured using a flow cytometer (FACS Aria, Becton Dickinson, NJ, USA). At least 10,000 events were acquired per sample. Data were analyzed using BD FACS DiVa software 2.1, and mean fluorescence intensity (MFI) was reported. Results were analyzed using Repeated-measures one-way ANOVA and Bonferroni test. Significance was set at *P* < 0.05.

## Results

### *In silico* identification of a TIR domain-containing thioredoxin in *E. histolytica*

We generated a HMM from a seed alignment (Pfam:PF01582) of TIR domain and searched proteins containing a TIR domain in Pathema database for *Entamoeba* genus. Two hypothetical proteins (Pathema:EIN_052840, E value = 0.00039; and Pathema:EIN_134280, E value = 0.00047) and two putative proteins (Pathema:EIN_092020, E value = 0.00047; and Pathema:EIN_191870, elongation factor 1-alpha, E value = 0.0016) were identified. A exploration on *Entamoeba histolytica* Protein databases for homologues TIR-domain containing proteins of any *Entamoeba* genus led us to identify a sequence which also contain a thioredoxin domain (GenPept:XP_649779). This whole sequence will be termed “thioredoxin” henceforth.

**Figure 1:**
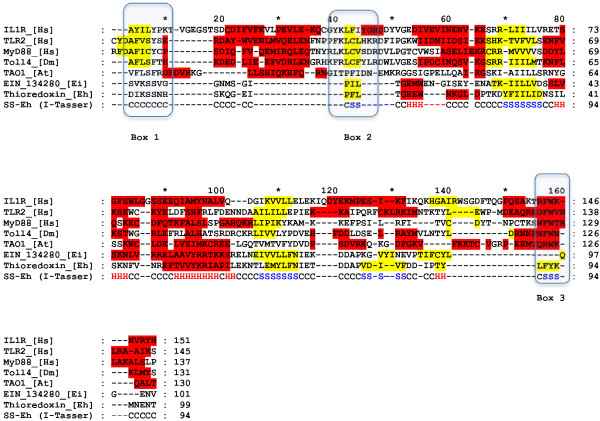
**Sequence alignment of TIR domains from several species.** Representative sequences are show from: *Homo sapiens* (IL-1R, TLR2, and MyD88_[Hs]), *Drosophila melanogaster* (Toll4_[Dm]), *Arabidopsis thaliana* (TAO1_[At]) as well as the sequences from *Entamoeba invadens* (EIN_134280_[Ei]) and *Entamoeba histolytica* (thioredoxin_[Eh]). Colors correspond to secondary structure derived from 3D structures for representative TIR domains, Psipred predictions for EIN_134280_[Ei] and the 3D modeled structure for Thioredoxin_[Eh]: helixes are colored in red and sheets in yellow. The three highly conserved regions previously described in TIR domains [14] are highlighted as boxes 1 to 3. I-Tasser secondary structure assignments are shown at the bottom (H= helix, S= sheet).

The putative TIR domain spanned positions 344–442, and its primary structure had low similitude with representative TIR domains from different proteins from different species, including human TLR2, IL1-R, and MyD88, *D. melanogaster* Toll4, and *A. thaliana* TAO1 (Figure 1). Even the Box1 and Box2, have structural coincidences, the Box3 exhibited differences in primary (absence of tyrosine residues) and secondary structure (presence of sheets, yellow colored, rather than helixes, red colored).

**Figure 2:**
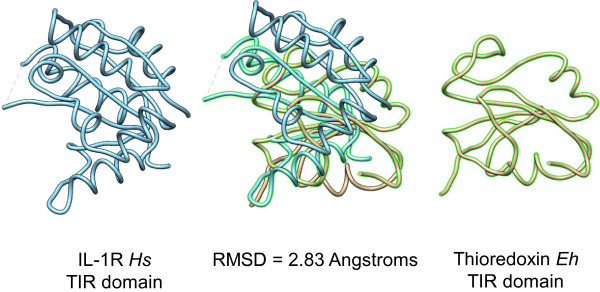
**Comparison of structures of IL1R from *Homo sapiens *and Thioredoxin from *E. histolytica *.** TIR domain of human interleukin-1 receptor (PDB: 1T3GA) is show in left model. Tertiary structure of TIR domain-containing thioredoxin was calculated by I-Tasser and show in right model. Structure superimposition is show in the center. (Calculated by Chimera).

To demonstrate that the region spanning positions 344 to 442 of this *E. histolytica* thioredoxin is a member of the TIR family, the 3D protein structure was modeled using a threading strategy on the I-Tasser server (Figure 2, right model). The specific one-on-one 3D structure alignment of the thioredoxin protein sequence with the human Inteleukin-1 Receptor (IL-1R) (Figure 2, left model) and TLR2 (data not shown) TIR domain sequences were determined. These alignments have a root-mean-square distance (RMSD) value of 2.83 and 6.1 Å, respectively, which corresponds to the similarity degree of the tridimensional structure of these proteins and suggests that this region is part of the TIR domain family. Accordingly, the 3D model of the TIR protein from *E. histolytica* was remarkably similar to the TIR domain (Figure 2, superimposition).

### Silencing of TIR domain-containing thioredoxin decrease the phagocytosis of human erythrocytes and *E. coli* by *E. histolytica* trophozoites

**Figure 3:**
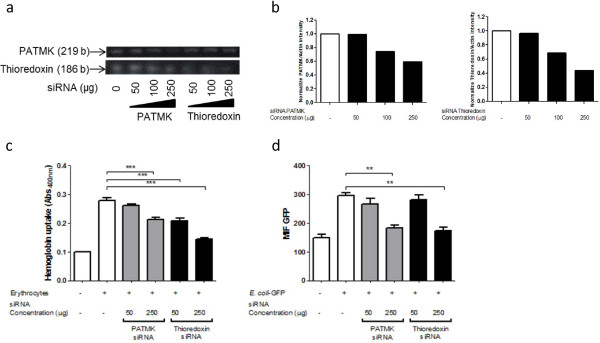
**Thioredoxin silencing in *E. histolytica *reduce the Erythrocytes and *E. coli *phagocytosis. ***Entamoeba histolytica* trophozoites were preincubated in the presence or absence of 50, 100 or 250 μg of PATMK and thioredoxin siRNA during 15 h. **a**) representative RT-PCR bands of PAMTK and thioredoxin amplified products of silencing and non-silencing trophozoites. **b**) Densitometric analysis RT-PCR products normalized to PATMK/Actin or Thioredoxin/Actin density of non-silenced trophozoites. **c**) Erythrophagocytosis of siRNA PATMK and thioredoxin silenced trophozoites. **d**) GFP-labeled *E. coli* phagocytosis of siRNA PATMK or thioredoxin silenced trophozoites (MIF: Median Intensity Fluorescence). Results were analyzed using Repeated-measures one-way ANOVA and Bonferroni test. Significance was set at *P* < 0.05.

To show the presence of the region of the mRNA corresponding to TIR domain on thioredoxin transcript, we designed a set of primers which amplified the 344 to 442 region corresponding to thioredoxin protein sequence. We found that *E. histolytica* trophozoites expressed the TIR domain-containing thioredoxin mRNA, as assessed using RT–PCR (Figure 3a).

To elucidate the possible participation of TIR domain-containing thioredoxin in phagocytosis process of erythrocytes and bacteria, we silenced the expression of thioredoxin putative mRNA in *E. histolytica* trophozoites using small interfering double stranded-RNAs (siRNA) ( Additional file 1: Table S1) and evaluated the silencing efficiency using RT–PCR. As a positive control of silencing, we also dowregulated the expression of the mRNA of PATMK protein, which has been involve in the initialization of erythrocytes phagocytosis in *E. histolytica* trophozoites, according to Boettner *et al.* study. This protein is a good candidate to repressed the phagocytosis process, due the inhibited by three pathways including monoclonal antibodies, mRNA knock-down using shRNA and PATMK gene mutation the reduction on erythrophagocytosis is clear [23].

We observed that 15 hours after the exposition to 250 μg of siRNA, the expression of the PATMK mRNA was reduced until ~40%, and the thioredoxin mRNA was reduced by ~60% (Figure 3a and 3b). According to literature, the downregulation of PATMK gene expression decreases the capability to phagocyte erythrocytes in trophozoites [23]. In other hand, trophozoites with reduced expression of thioredoxin also exhibited less ability to ingest erythrocytes in a concentration-dependent fashion (Figure 3c). In the same way, downregulation of thioredoxin transcripts, also decreases the capability of trophozoites to engulf *E. coli*-GFP compared with untreated trophozoites (Figure 3d).

Previously, Boettner *et* al. [23], demonstrated that PATMK silencing decrease the erythrocytes phagocytosis, and in this report we show that PATMK is also required for the bacteria phagocytosis (Figure 3d). All these results suggest that the TIR domain-containing thioredoxin, similar to PATMK, could be associated to a phagocytosis process on *E. histolytica* trophozoites.

## Discussion

The TIR domain consists of approximately 200 amino acids that form five β sheets surrounded by five α helixes [14]. In animals and plants, as well as in free-living amebae, the TIR domain participates in diverse biological activities such as pathogen resistance, immune recognition and feeding [13,24-27]. In this study, we identified a thioredoxin in the *E. histolytica* proteome which contains a putative TIR domain in a thioredoxin protein. Primary structure of this TIR domain differ from other TIR domains found in plants and animals, but secondary structures show more similar regions, mainly in Box1 and Box2 that are essential regions to fold the protein, and both are conformed by two helixes [14]. On the other hand, the specific one-on-one 3D structure alignment showed that the thioredoxin has structural analogues of TIR domains in mammalian protein IL-1R and TLR2. This supports the idea that three-dimensional structure is much more closely associated with function than its lineal sequence, tertiary structure is more evolutionarily conserved than primary structure ( http://www.ncbi.nlm.nih.gov/books/NBK22362). Both TIR domains, of mammalian IL-1R and TLR2, mediate homotypic protein–protein interactions in the signal transduction that facilitate the activation and expression of mechanisms which increase several biological process, including phagocytic activity [14,28,29].

The participation of this domain on phagocytosis during infection resistance and feeding observed in the evolutionary related ameba *Dictyostelium discoideum*[13,15,30], led us to think that the thioredoxin found, may participate in the phagocytosis of natural sources of nutrients through human commensal bacteria (such as *Escherichia coli*) and erythrocytes to uptake iron [23]. In *E. histolytica*, phagocytosis process is Gal/GalNAc lectin and PATMK dependent [31].

In this report, we observed reduction in phagocytosis of human erythrocytes and *E. coli* after incubation with small interfering double stranded-RNAs for thioredoxin, suggesting a role for the TIR domain-containing thioredoxin in trophozoites phagocytosis. These results could support the proteomic approach explored by McCoy *et al.*, and Okada *et al.*[32,33], which suggest that the phagocytosis process Gal/GalNAc lectin-mediated involves the recruitment of thiol-specific antioxidants (such as thioredoxin) for phagosome biogenesis and cytoskeletal rearrangement [8-10]. If this is right, PATMK and Gal/GalNAc lectin, both of which are involved in the interaction with the membrane of erythrocytes, could be involved in the formation of microdomains necessaries to signaling in phagocytosis, in which molecules like thioredoxin could participate. However this must be investigated further.

The above mentioned, is similar to reported to the majority of these TIR domain-containing proteins that have been described in plants, insects, and vertebrates and participate in phagocytic and cell activation [13,25]. However, despite finding similarities among the reduction of phagocytosis when the expression of these transcripts is reduced, according to other reports, we cannot reject the possibility that the thioredoxin identified here participates in the phagocytic process via a mechanism that is not related to its TIR domain, for example the redox state thioredoxin-dependent processes which regulate cellular functions, such as endocytosis and cell adhesion [34-36].

A diverse number of specific functions and cofactor activities have been described for thioredoxins. These functions include growth factor activity, cell protection from toxic compounds (especially oxidants and electrophiles), activation of inflammatory pathways, regulation of transcription factors and protein binding, and catalysis of protein folding; each of these activities has an effect on cellular responses to toxic insults [11,12]. However, no other thioredoxin is involved in the regulation of phagocytic processes. *E. histolytica* expresses other proteins with thioredoxin activity that participate in nicotinamide adenine dinucleotide phosphate (NADPH)-dependent hydrogen peroxide reduction [35]; these detoxifying thioredoxins do not share the TIR domain identified here.

In this report we present evidence that protein sequence corresponding to 344–442 position in *E. histolytica* thioredoxin (GenPept:XP_649779) has a TIR-domain. Considering that several members of TIR domain family have been involved in signal transduction in many organisms, and silencing its expression decrease erythrocytes and bacteria phagocytosis, the thioredoxin could play a central role in signal transduction during this event, hence influencing the *E. histolytica* survival mechanisms.

## Conclusion

Uptake of nutrients in *Entamoeba histolytica* by phagocytosis involves recruitment of proteins such as PATMK, and our results suggest that TIR domain-containing thioredoxin also can support this activity on bacterial and erythrocytes phagocytosis.

## Competing interests

The authors have declared that no competing interests exist.

## Authors’ contributions

IMH, AMT, AJU, IWB, and CLM conceived the study, and all authors participated in the study design, coordination and writing the manuscript. All authors read and approved the final manuscript.

## Supplementary Material

Additional file 1 **Table S1.** Double-stranded oligonucleotides used for the silencing of the expression of thioredoxin and PATMK in *E. histolytica* trophozoites. Click here for file
