# Supplementary material for: A Toll/IL-1R/resistance domain-containing thioredoxin regulates phagocytosis in Entamoeba histolytica
Source: Parasit Vectors. 2012 Oct 8;5:224. doi: 10.1186/1756-3305-5-224 (PMC3481431; doi:10.1186/1756-3305-5-224)
Supplement: Additional file 1 — Table S1. Double-stranded oligonucleotides used for the silencing of the expression of thioredoxin and PATMK in E. histolytica trophozoites. [file 1756-3305-5-224-S1.pdf]

Supplementary

Table S1. Double-stranded oligonucleotides used for the silencing of the expression of thioredoxin and PATMK in *E. histolytica* trophozoites.

Transcript target	Sequence and characteristics
Thioredoxin	5' rGrGrCrArCrUrUrGrArArGrUrGrGrCrUrGrArArArUrGrU G T<br>rUrArCrCrGrUrGrArArCrUrUrCrArCrCrGrArCrUrUrUrArCrArCrA 5'<br>Sense Position 123 AntiSense Position: 147<br>Sense Length: 25 AntiSense Length: 27<br>Sense MW: 7986.9 AntiSense MW: 8539.1<br>Total MW: 16526.0 Core GC%: 36.8
	5' rGrGrArArUrArArArGrGrGrUrUrArGrArCrCrCrArArCrA A A<br>rUrArCrCrUrUrArUrUrUrCrCrCrArArUrCrUrGrGrGrUrUrGrUrUrU 5'<br>Sense Position: 991 AntiSense Position: 1015<br>Sense Length: 25 AntiSense Length: 27<br>Sense MW: 8067.0 AntiSense MW: 8447.0<br>Total MW: 16514.0 Core GC%: 47.4
	5' rGrArArCrCrUrUrCrUrArCrArUrCrUrArCrArArCrArUrU T A<br>rUrUrCrUrUrGrGrArArGrArUrGrUrArGrArUrGrUrUrGrUrArArArU 5'<br>Sense Position: 1635 AntiSense Position: 1659<br>Sense Length: 25 AntiSense Length: 27<br>Sense MW: 7863.7 AntiSense MW: 8626.2<br>Total MW: 16490.0 Core GC%: 36.8
PATMK	5' rGrCrArUrUrUrCrArGrCrCrArUrUrArCrCrUrCrCrArArU A G<br>rArUrCrGrUrArArArGrUrCrGrGrUrArArUrGrGrArGrGrUrUrArUrC 5'<br>Sense Position: 116 AntiSense Position: 140<br>Sense Length: 25 AntiSense Length: 27<br>Sense MW: 7821.7 AntiSense MW: 8690.3<br>Total MW: 16512.0 Core GC%: 36.8
	5' rArGrArArGrArUrGrCrArGrArUrGrArArUrUrUrGrUrUrA A T<br>rUrUrUrCrUrUrCrUrArCrGrUrCrUrArCrUrUrArArArCrArArUrUrA 5'<br>Sense Position: 1996 AntiSense Position: 2020<br>Sense Length: 25 AntiSense Length: 27<br>Sense MW: 7991.8 AntiSense MW: 8538.2<br>Total MW: 16530.0 Core GC%: 42.1
	5' rGrArCrArCrArGrCrArGrGrUrCrArArGrArArArGrGrUrU T A<br>rUrArCrUrGrUrGrUrCrGrUrCrCrArGrUrUrCrUrUrUrCrCrArArArU 5'<br>Sense Position: 3062 AntiSense Position: 3086<br>Sense Length: 25 AntiSense Length: 27<br>Sense MW: 8117.0 AntiSense MW: 8312.9<br>Total MW: 16429.9 Core GC%: 31.6
